# Myocardial injury in critically ill patients admitted with noncardiac diagnoses

**DOI:** 10.1186/cc12172

**Published:** 2013-03-19

**Authors:** J Lo, K Lei, I Webb, J Coutts, J Chambers, A Griffiths, J Smith, E Connell, P Collinson, J Peacock, D Treacher, M Ostermann

**Affiliations:** 1 King's College London, London, UK; 2 Guys & St Thomas Foundation Hospital, London, UK; 3 St George's Hospital, London, UK

## Introduction

In the critically ill, the incidence of raised cardiac troponin T (cTnT) levels is high. Although the mechanisms of myocardial injury are not well understood, raised cTnT levels are associated with increased mortality. The aim of our study was to determine the incidence, prevalence and outcome of silent myocardial injury as determined by raised cTnT levels and concomitant ECG changes in critically ill patients admitted for noncardiac reasons.

## Methods

ECGs were taken and cTnT was measured daily during the first week and on alternate days during the second week until discharge from the ICU or death. After completion of the study, all cTnT levels and ECGs were analysed independently and patients were classified into four groups: definite MI (cTnT ≥15 ng/l and definite ECG changes of MI), possible MI (cTnT ≥15 ng/l and ischaemic changes on ECG), troponin rise alone (cTnT ≥15 ng/l with no ischaemic ECG changes), or normal. All medical notes were reviewed independently by two ICU clinicians.

## Results

A total of 144 patients were included in the analysis (42% female; mean age 61.9 (SD 16.9); mean APACHE II score 19.4). In total, 121 patients (84%) had at least one cTnT level ≥15 ng/l during their stay in the ICU. Twenty patients (14%) fulfilled criteria for a definite MI, of whom 65% were septic and 50% were on noradrenaline at the time (ICU and hospital mortality: 25% and 30%, respectively). Thirty-nine patients (27%) had a possible MI, of whom 69% were septic and on noradrenaline (ICU and hospital mortality: 31% and 41%, respectively). Sixty-two patients (43%) had a raised troponin without ECG, of whom 69% were septic and 50.7% were on noradrenaline (ICU and hospital mortality: 23% and 31%, respectively). Twenty-three patients had normal cTnT results and serial ECGs, of whom 61% had sepsis. ICU and hospital mortality was 4%. Only 25% of definite MIs and 18% of possible MIs were recognised by the clinical teams at the time.

## Conclusion

Eighty-four per cent of critically ill patients had a raised cTnT level at some stage during their stay in the ICU. More than 40% of patients fulfilled criteria for a possible or definite MI, of whom only 20% were recognised clinically. ICU and hospital outcome were significantly worse in patients with a cTnT rise. The proportion of patients with sepsis was similar between the patients with a definite, possible or no MI.

